# Nickel-Catcher-Doped Zwitterionic Hydrogel Coating on Nickel–Titanium Alloy Toward Capture and Detection of Nickel Ions

**DOI:** 10.3389/fbioe.2021.698745

**Published:** 2021-06-24

**Authors:** Xiaoyi Fu, Xi Liu, Dezhao Hao, Wuyi Xiao, Qiong Nie, Jingxin Meng

**Affiliations:** ^1^National Center of Stomatology, National Clinical Research Center for Oral Diseases, National Engineering Laboratory for Digital and Material Technology of Stomatology, Department of Orthodontics, Peking University School and Hospital of Stomatology, Beijing, China; ^2^CAS Key Laboratory of Bio-Inspired Materials and Interfacial Science, CAS Center for Excellence in Nanoscience, Technical Institute of Physics and Chemistry, Chinese Academy of Sciences, Beijing, China; ^3^University of Chinese Academy of Sciences, Beijing, China

**Keywords:** nickel ion capture, anti-bacteria adhesion, zwitterionic hydrogel, surface modification, nickel–titanium alloy

## Abstract

Nickel–titanium (NiTi) alloys show broad applicability in biomedical fields. However, the unexpected aggregation of bacteria and the corrosion of body fluid on NiTi-based medical devices often lead to the leakage of nickel ions, resulting in inevitable allergic and cytotoxic activities. Therefore, the capture and detection of nickel ions are important to avoid serious adverse reactions caused by NiTi-based medical devices. Herein, we presented a nickel ion capture strategy by the combination of zwitterionic hydrogels as anti-bacteria layers and carbon disulfide (CS_2_) components as nickel-catchers (Ni-catchers). On the one hand, the hydration layer of zwitterionic hydrogel can efficiently inhibit bacteria adhesion and reduce nickel ions leakage from NiTi corrosion. On the other hand, Ni-catchers can capture leaked nickel ions from NiTi alloy actively by chelation reaction. Therefore, this strategy shows great capabilities in resisting bacteria adhesion and capturing nickel ions, providing the potential possibility for the detection of nickel ion leakage for implantable biomedical materials and devices.

## Introduction

With the rapid development of biomedicine, implantable materials have been widely used in diagnosis, treatment, and prosthesis (Sylvestre et al., 2020). As one of the most popular biomaterials, nickel–titanium (NiTi) alloys have been applied in orthodontic archwires (Liu et al., 2019), bone implants (Raphel et al., 2016), and vascular stents (Robertson and Ritchie, 2007), due to their unique superelasticity and good biocompatibility. However, the leakage of nickel ions (Ni^2+^ ions) from NiTi alloys often leads to adverse reactions such as allergic signs (e.g., contact mucositis and eczematic and urticarial reactions; Huang et al., 2003; Kolokitha and Chatzistavrou, 2008) and cytotoxic responses (Jia et al., 1999). In addition, the unexpected adhesion of bacteria and acidic oral environment corrode NiTi alloys, aggravating the leakage of Ni^2+^ ions (Huang et al., 2003). Therefore, a novel strategy for capturing and detecting leaked Ni^2+^ ions is urgently needed for potential biomedical applications.

In recent years, many passive strategies have been developed to avoid the leakage of Ni^2+^ ions, including growing oxide films [e.g., TiO_2_ layer (Gu et al., 2005) and NiTiO_3_ nanosheet (Hang et al., 2019)], depositing coatings [e.g., diamond-like carbon coating (Hang et al., 2012) and 1*H*,1*H*,2*H*,2*H*-perfluorodecyltrimethoxysilane (FAS) deposited layer (Liu et al., 2019)], and implanting barrier elements [e.g., carbon (Poon et al., 2005) and oxygen (Wu et al., 2006) plasma immersion ion implantation]. However, most of them can not prohibit long-term leakage of Ni^2+^ ions because of the low stability of hydrophobic surface in acidic oral environment (Liu et al., 2019) and the corrosion of adhered bacteria (Gu et al., 2005). Moreover, there are many anti-bacteria methods including zwitterionic hydrogels (Li et al., 2008; Shivapooja et al., 2015; Lin et al., 2020), polyethylene glycol (Peng et al., 2017), quaternary ammonium salt groups (Wei et al., 2016, 2017), protein-based surfaces (Liu et al., 2018), and multiwalled carbon nanotubes (Hartono et al., 2018). Thus, combining anti-bacteria and Ni^2+^ ion capture may be a promising strategy for the new generation of NiTi-based medical devices. As a conventional medical treatment for acute carbonyl nickel poisoning, carbon disulfide (CS_2_) derivates can serve as Ni-catchers by chelation reaction (Sunderman, 1990). Therefore, we believe that introducing Ni-catchers into anti-bacteria layer can achieve long-term Ni^2+^ ion capture and detection.

**FIGURE 1:**
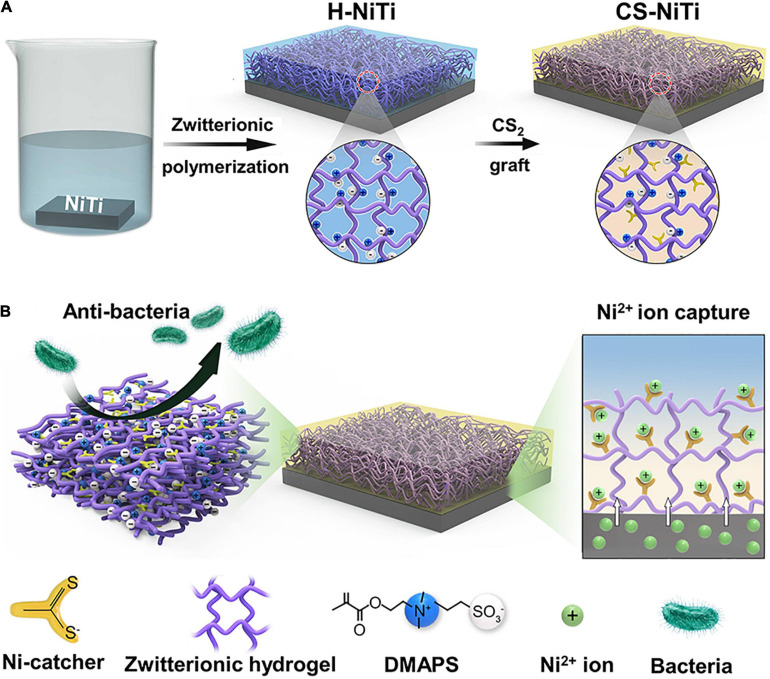
Design principle of CS-NiTi for long-term Ni^2+^ ion capture. **(A)** Schematic of the fabrication process of CS-NiTi. **(B)** The as-prepared CS-NiTi shows dual functions of anti-bacteria and Ni^2+^ ion capture.

Herein, we present a Ni-catcher-doped zwitterionic hydrogel coating on the surface of NiTi alloy, showing robust anti-bacteria and long-term Ni^2+^ ion capture (Figure 1). In brief, zwitterionic hydrogel was firstly prepared on the surface of NiTi alloy (H-NiTi) by the copolymerization of 2-hydroxyethyl methacrylate (HEMA), 3-dimethyl(methacryloyloxyethyl)ammonium propane-sulfonate (DMAPS), and methyl methacrylate (MMA; Su et al., 2020). Then, the CS_2_ as a Ni-catcher was grafted into zwitterionic hydrogel by immersing H-NiTi in the mixture of CS_2_ and triethylamine (TEA) to obtain CS-NiTi (Figure 1A and Supplementary Figure 1). The zwitterionic hydrogel can prevent the adhesion of bacteria by the hydration layer on the surface of CS-NiTi. Moreover, the Ni-catcher endows this coating with Ni^2+^ ion capture through chelation reaction. Therefore, this work provides a promising avenue to capture and detect Ni^2+^ ions, which could be used for implantable biomedical materials and devices.

## Materials and Methods

### Chemicals and Materials

Nickel–titanium alloy was purchased from Sunway Technology Co. Ltd. (Guangdong, China). Brain heart infusion (BHI) was obtained from Oxoid Co. (Hampshire, United Kingdom). Dulbecco’s modified Eagle medium (DMEM) was bought from Thermo Fisher Biochemical Products Co. Ltd. (Beijing, China). Tris–HCl buffer was purchased from Coolaber Science Technology (Beijing, China). Phosphate-buffered saline (PBS) was bought from HyClone Laboratories Inc. (Logan, UT, United States). Acetone (>99.5%, AR) and alcohol (≥99.8%, GR) were obtained from the Beijing Chemical Co. (Beijing, China). Bis(2-methacryloxyethyl)phosphate (bis-HEMAP) and 2,2-diethoxyacetophenone (DEAP) were bought from J&K Scientific (Beijing, China). HEMA and DMAPS were purchased from Aladdin Ltd. (Shanghai, China). MMA and poly(ethylene glycol)dimethacrylate (PEGDMA) were purchased from Alfa Aesar (Haverhill, MA, United States). CS_2_ and TEA were obtained from Macklin Inc. (Shanghai, China). Deionized water (>1.82 MΩ cm, Milli-Q system, Merck & Co., Kenilworth, NJ, United States) was used. All reagents were used as received.

### Preparation of CS-NiTi

The CS-NiTi was fabricated according to previous literature, with some modifications (Su et al., 2020). Firstly, pristine NiTi alloy was immersed in bis-HEMAP (5% ethanol solution) for 24 h and washed with deionized water for three times. Then, the 0.5 wt% of the photoinitiator DEAP was coated on the surface of NiTi alloy. The hydrogel precursor solution was obtained by mixing the crosslinker of PEGDMA (2 wt%) with the hydrogel monomers solution, including DMAPS (5 wt%), HEMA (40 wt%), and MMA (5 wt%). Then, it was degassed for 20 min by KQ5200DE ultrasonic cleaner (Kun Shan Ultrasonic Instruments Co. Ltd., Shanghai, China). The degassed precursor solution was poured on the surface of NiTi alloy and exposed to UV irradiation (wavelength ≈ 365 nm) in a dark chamber for 600 s by a UV LED curing system (Beijing NBET Technology Co. Ltd., Beijing, China), to obtain H-NiTi. To graft Ni-catchers on H-NiTi, H-NiTi was immersed into a mixture for 24 h containing CS_2_, TEA, and deionized water. LCS-NiTi, MCS-NiTi, and HCS-NiTi were obtained by regulating the concentration of CS_2_ (0.2, 0.6, and 1.0 mol/L), respectively.

### Characterizations

The morphology of pristine NiTi, H-NiTi, and CS-NiTi were observed with S-4800 scanning electron microscope (SEM; Hitachi, Tokyo, Japan) and Single Lens Reflex camera (SLR camera; Nikon D80, Tokyo, Japan). Water contact angles (WCAs) were measured by dropping 2 μl of water on the surface of pristine NiTi, H-NiTi, and CS-NiTi by the Dataphysics OCA 20 Contact-Angle System (Filderstadt, Germany). The S element analysis was performed on ESCALAB 250Xi X-ray photoelectron spectroscopy analysis (XPS; Thermo Fisher Scientific, Waltham, MA, United States). The transmittance was measured by UV-2600 UV-VIS spectrophotometer (SHIMADZU, Kyoto, Japan) with wavelength from 450 to 700 nm.

### Cell Culture and Cellular Toxicity Test

Cell Counting Kit-8 (CCK-8) assay was performed to investigate the cell viability (Xiao et al., 2020). And the cytotoxicity of materials including pristine NiTi, H-NiTi, and CS-NiTi was evaluated with HepG2 cells. HepG2 cells with the density of 1 × 10^5^ cells per well were seeded into the 24-well plates in DMEM containing 10% fetal bovine serum (FBS) and 1% antibiotic solution (penicillin and streptomycin) and then cultured at 37 C in a humidified atmosphere with 5% CO_2_ for 36 h. Pristine NiTi, H-NiTi, and CS-NiTi were added to each well, and the cells were incubated for 12 h. Subsequently, CCK-8 solutions were added to the wells followed by 2-h incubation at 37°C. And then the supernatant was obtained and added to 96-well plates. Finally, microplate reader was used to measure the absorbance values per well at a test wavelength of 450 nm and a reference wavelength of 690 nm to analyze the cell viability. Control experiments were done under the same condition with PBS. Eq. (1) was used to calculate the cell viability rate:

(1) Cellviability=As/Ac×100%

where *As* represents the absorbance of the treatments of different groups and *Ac* represents the absorbance of the control treatments with PBS. All the experiments were performed in triplicate.

### Bacteria Cultivation

*Streptococcus mutans* (UA159) was used as the model bacteria. *S. mutans* was revived by incubating its freezing solution with 1 ml of BHI at 37°C for 48 h. Then the suspension of *S. mutans* was centrifugated at 2,000 rpm for 10 min at room temperature to discard the supernatant. Then, the *S. mutans* aggregation was resuspended in another 1 ml of BHI and incubation at 37°C for another 48 h. After the logarithmic phase was reached, the bacteria were harvested by centrifugation at 2,000 rpm for 10 min at room temperature and washed with Tris–HCl buffer three times. Subsequently, the bacteria were suspended in 50 ml of Tris–HCl buffer at a final concentration of 1 × 10^8^ CFU/ml (Liu et al., 2018).

### Anti-bacteria Property

To test anti-bacteria ability, the pristine NiTi, H-NiTi, and CS-NiTi were incubated in the as-prepared bacteria suspensions for 1, 3, 6, 12, 18, and 24 h at 37°C. After the incubation, they were taken out and fixed in 2.5% glutaraldehyde for 1 h at room temperature, followed by immersing in a graded series of alcohol concentrations (30, 50, 70, 80, 90, and 100%) for 10 min to dehydrate the adhered bacteria. SEM was used to observe bacteria adhesion performance. The quantitative evaluation of the adhered bacteria was obtained by Cell Profiler 4.0.7. The anti-bacteria efficiency (*E_anti–bacteria_*) could be calculated by Eq. (2):

(2) $$E_{anti-bacteria}=\left(1-D_{CS-NiTi}/D_{NiTi}\right)\times 100\%$$

where *D_CS–NiTi_* and *D_NiTi_* refer to the bacteria density on the surface of CS-NiTi and pristine NiTi, respectively.

### Performance of Ni^2+^ Ion Capture

The performance of Ni^2+^ ion capture was investigated on 710-OES inductively coupled plasma emission spectrometer (ICP; Varian, Palo Alto, CA, United States) by detecting the concentration of nickel ions in the supernatant of the remaining bacteria suspension incubated with pristine NiTi, H-NiTi, LCS-NiTi, MCS-NiTi, and HCS-NiTi for different times at 37°C. The capture efficiency of Ni^2+^ ions (*E_capture_*) could be calculated by Eq. (3):

(3) $$E_{capture}=\left(1-C_{CS-NiTi}/C_{H-NiTi}\right)\times 100\%$$

where *C_CS–NiTi_* and *C_H–NiTi_* refer to the concentration of Ni^2+^ ions in the supernatant of remaining bacteria suspension incubated with CS-NiTi and H-NiTi, respectively.

## Results

### Characterizations of CS-NiTi

**FIGURE 2:**
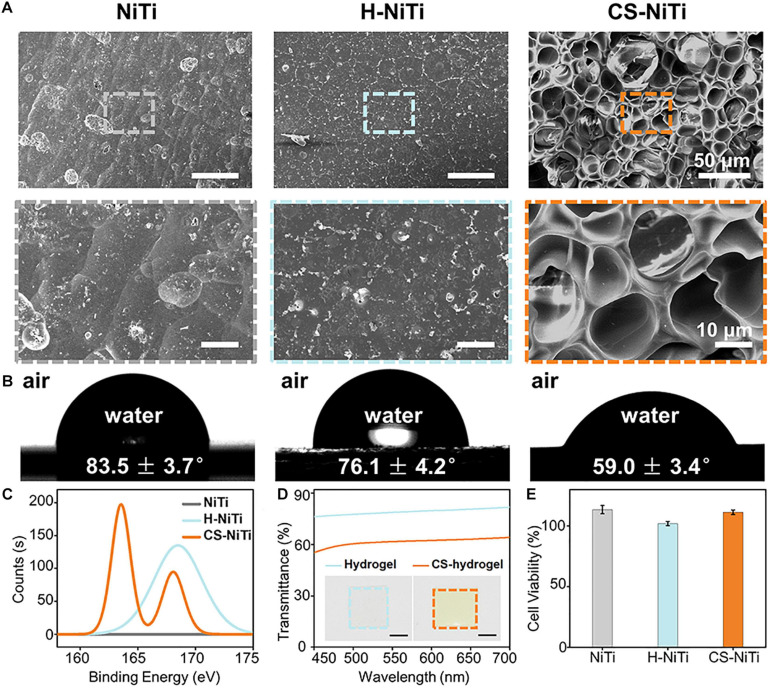
Characterizations of CS-NiTi. SEM images **(A)**, water contact angles (WCAs; **B)**, X-ray photoelectron spectroscopy (XPS) spectra **(C)**, transmittance, **(D)** and biocompatibility **(E)** of pristine NiTi, H-NiTi, and CS-NiTi. The insets in **(D)** show the optical images of zwitterionic hydrogel and CS-hydrogel. Scale bar, 5 mm for **(D)**.

To verify the introduction of Ni-catchers, we compared the pristine NiTi, H-NiTi, and CS-NiTi from surface morphology, WCAs, XPS spectra, transmittance, optical images, and biocompatibility. As shown in Figure 2A, the introduction of Ni-catchers resulted in the porous morphology of CS-NiTi, compared with the flat morphology of H-NiTi, probably because of the phase separation (Su et al., 2020). Furthermore, the WCAs decreased from 83.5 ± 3.7° (NiTi) to 59.0 ± 3.4° (CS-NiTi), indicating the hydrophilicity of CS-NiTi (Figure 2B). The change of wettability might be attributed to the formation of hydrophilic xanthates in the presence of TEA (Weißl et al., 2018) and the porous morphology of CS-NiTi. More hydrophilic surface was beneficial to the formation of the hydration layer, enhancing the anti-bacteria adhesion ability of NiTi alloys (Lin et al., 2020). XPS was further employed to verify the presence of Ni-catchers in CS-NiTi. As shown in Figure 2C and Supplementary Figure 2, two S 2p peaks at 163 and 168 eV were observed in the XPS spectrum of CS-NiTi, compared with the single S 2p peak at 168 eV for H-NiTi and no S 2p peak for NiTi. The S 2p peak at 168 eV shown in H-NiTi represented the S element in zwitterionic hydrogel network. The new S 2p peak at 163 eV indicated the successful introduction of Ni-catchers in CS-NiTi (Deng et al., 2013). Moreover, the appearance of zwitterionic hydrogels changed from colorless to yellow with lower transmittance after Ni-catcher (CS_2_) graft (Figure 2D), for xanthates always appear as a series of yellow compounds. The transmittance of Ni-catcher-doped zwitterionic hydrogel coating was higher than 60%, which has an insignificant impact on the appearance of NiTi alloys and would not affect the application of NiTi alloys in the implantable biomedical field. Furthermore, we tested the biocompatibility of CS-NiTi using CCK-8 test (Xiao et al., 2020). Higher HepG2 cell viability in Figure 2E indicated the excellent biocompatibility of CS-NiTi, showing promising applicability in biomedical field. Therefore, we successfully fabricated CS-NiTi with good biocompatibility.

### Anti-bacteria Property of CS-NiTi

**FIGURE 3:**
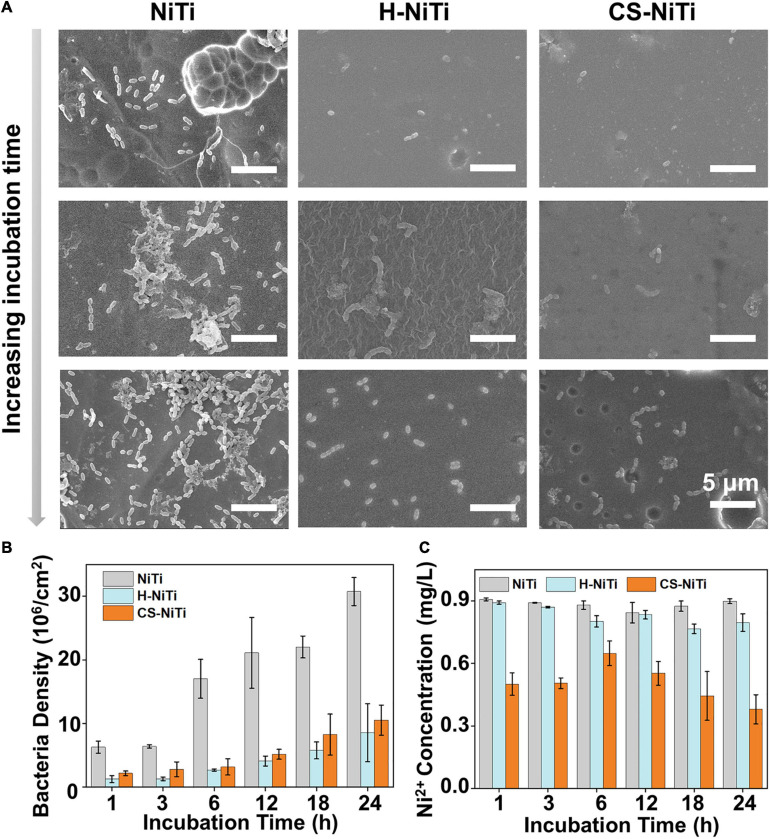
The anti-bacteria property of CS-NiTi. **(A)** SEM images of pristine NiTi, H-NiTi, and CS-NiTi after being incubated with bacteria suspension for different times (1, 12, and 24 h). The quantitative analysis of bacteria adhesion **(B)** and Ni^2+^ ion capture performance **(C)** on the surface of pristine NiTi, H-NiTi, and CS-NiTi at different incubation times.

As a kind of widely used orthodontic material, NiTi alloys are often exposed to the oral environment. However, cleaning teeth is difficult during orthodontic treatment, leading to the adhesion of bacteria and causing caries and periodontitis. To investigate the anti-bacteria property in the oral environment, CS-NiTi was incubated in 1 × 10^8^ CFU/ml bacteria suspension (*S. mutans*, the largest proportion of bacteria in oral environment) for different times, taking pristine NiTi and H-NiTi as controls. As shown in Figure 3A and Supplementary Figure 3, many bacteria adhered on the surface of pristine NiTi after being incubated for 1 h. In contrast, there were a few bacteria on the surfaces of H-NiTi and CS-NiTi, indicating their good anti-bacteria properties. By prolonging incubation time, a growing number of bacteria adhered on the surface of pristine NiTi and formed a biofilm at 12 h. In contrast, the bacteria density was still very low on the surfaces of H-NiTi and CS-NiTi. The quantitative analysis in Figure 3B revealed a good anti-bacteria efficiency in H-NiTi and CS-NiTi at 6 h, showing the highest efficiency of ca. 84% for H-NiTi and ca. 81% for CS-NiTi. By prolonging the incubation time to 24 h, the anti-bacteria efficiency maintained higher than 72% for H-NiTi and 66% for CS-NiTi. These results indicated that CS-NiTi had excellent long-term anti-bacteria property. Moreover, ICP was employed for exploring the performance of Ni^2+^ ion capture by monitoring the concentration of Ni^2+^ ions in bacteria suspension (1 × 10^8^ CFU/ml). As shown in Figure 3C, the concentration of Ni^2+^ ions in CS-NiTi was significantly lower than that of NiTi and H-NiTi. Even though it was incubated for 24 h, the concentration of Ni^2+^ ions for CS-NiTi was still as low as 0.38 ± 0.07 mg/L, indicating the excellent ability of Ni^2+^ ion capture of CS-NiTi. Therefore, CS-NiTi showed dual properties of anti-bacteria and Ni^2+^ ion capture at the same time.

### Mechanism of Ni^2+^ Ion Capture

**FIGURE 4:**
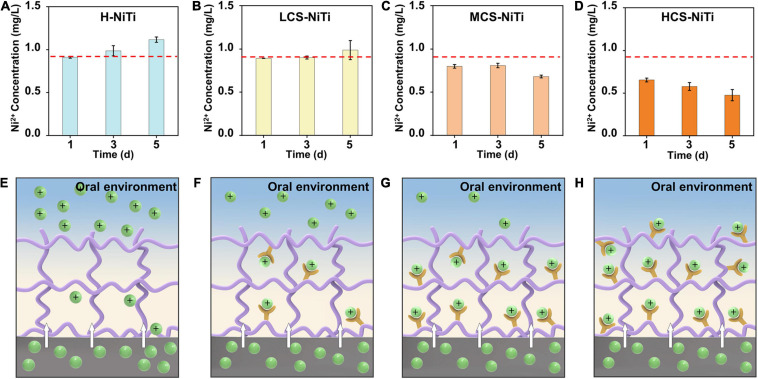
The mechanism of Ni^2+^ ion capture of CS-NiTi. **(A–D)** The quantitative analysis of Ni^2+^ ion capture performance of H-NiTi, LCS-NiTi, MCS-NiTi, and HCS-NiTi. **(E–H)** Schematic of Ni^2+^ ion capture property of CS-NiTi with different concentrations of Ni-catchers.

To further investigate the performance of Ni^2+^ ion capture, a series of CS-NiTi were fabricated by regulating the CS_2_ concentration in the mixed solution, that is, 0.2 mol/L of CS_2_ for low concentration (denoted as LCS-NiTi), 0.6 mol/L of CS_2_ for medium concentration (MCS-NiTi), and 1.0 mol/L of CS_2_ for high concentration (HCS-NiTi). With the increasing concentration of Ni-catchers, the surface became more hydrophilic, revealing by the WCAs changing from 73.0 ± 2.8° to 59.0 ± 3.4°, along with the increased pore size (Supplementary Figures 4A,B). Furthermore, the appearance of CS-hydrogel changed from colorless with high transparency to yellow with reduced transparency (Supplementary Figure 4C). To verify the long-term ability of Ni^2+^ ion capture, we incubated H-NiTi, LCS-NiTi, MCS-NiTi, and HCS-NiTi with *S. mutans* suspensions for different days. After the incubation, the concentration of leaked Ni^2+^ ions from NiTi alloy was reduced from 0.91 ± 0.01 mg/L (H-NiTi) to 0.65 ± 0.02 mg/L (HCS-NiTi) with the incubation time of 1 day (Figures 4A–D). By prolonging the incubation time to 5 days, the concentration of leaked Ni^2+^ ions increased to 1.12 ± 0.03 mg/L for H-NiTi gradually. On the contrary, the concentration of leaked Ni^2+^ ions in HCS-NiTi reduced from 0.65 ± 0.02 to 0.48 ± 0.07 mg/L dramatically, indicating its excellent long-term property of Ni^2+^ ion capture.

The possible mechanism of Ni^2+^ ion capture is proposed in Figures 4E–H. Due to the corrosion of the adhered bacteria and acidic oral microenvironment, Ni^2+^ ions are released slowly and persistently from NiTi alloy (Huang et al., 2003). Briefly, the leaked Ni^2+^ ions can pass through the zwitterionic hydrogel coating on the surface of NiTi alloy and be released to oral environment easily (Figure 4E), as the diffusion of Ni^2+^ ions is not efficiently restricted in single zwitterionic hydrogel. In contrast, the concentration of Ni^2+^ ions is dramatically decreased after introducing Ni-catchers because grafted CS_2_ can capture free Ni^2+^ ions and restrain their diffusion to oral environment (Figures 4F–H). Moreover, the long-term capture efficiency of Ni^2+^ ions is improved with the increasing concentration of Ni-catchers. Therefore, the introduction of Ni-catchers endows CS-NiTi with robust Ni^2+^ ion capture property.

## Discussion

In summary, we demonstrate a long-term Ni^2+^ ion capture coating on the surface of NiTi alloy. The zwitterionic hydrogels and Ni-catchers endow CS-NiTi with excellent anti-bacteria and Ni^2+^ ion capture properties. The CS-NiTi exhibited long-term anti-bacteria efficiency of ca. 66% at 24 h and Ni^2+^ ion capture efficiency of ca. 57% at 5 days. Furthermore, by integrating chelation reaction and introducing color change, this coating may apply to Ni^2+^ ion capture and detection. Therefore, this coating provides a new clue to design the next generation of biomaterials and devices with capture and detection of Ni^2+^ ions.

## Data Availability Statement

The raw data supporting the conclusions of this article will be made available by the authors, without undue reservation.

## Author Contributions

QN and JM conceived the idea. DH and XF contributed the fabrication process. XF and WX performed the experiments. XF and XL analyzed the results. XF, XL, QN, and JM wrote the manuscript. All authors contributed to the article and approved the submitted version.

## Conflict of Interest

The authors declare that the research was conducted in the absence of any commercial or financial relationships that could be construed as a potential conflict of interest.
