# Supplementary material for: Nickel-Catcher-Doped Zwitterionic Hydrogel Coating on Nickel–Titanium Alloy Toward Capture and Detection of Nickel Ions
Source: Front Bioeng Biotechnol. 2021 Jun 24;9:698745. doi: 10.3389/fbioe.2021.698745 (PMC8264594; doi:10.3389/fbioe.2021.698745)
Supplement: Supplementary file 1 [file Data_Sheet_1.docx]

Supplementary Material


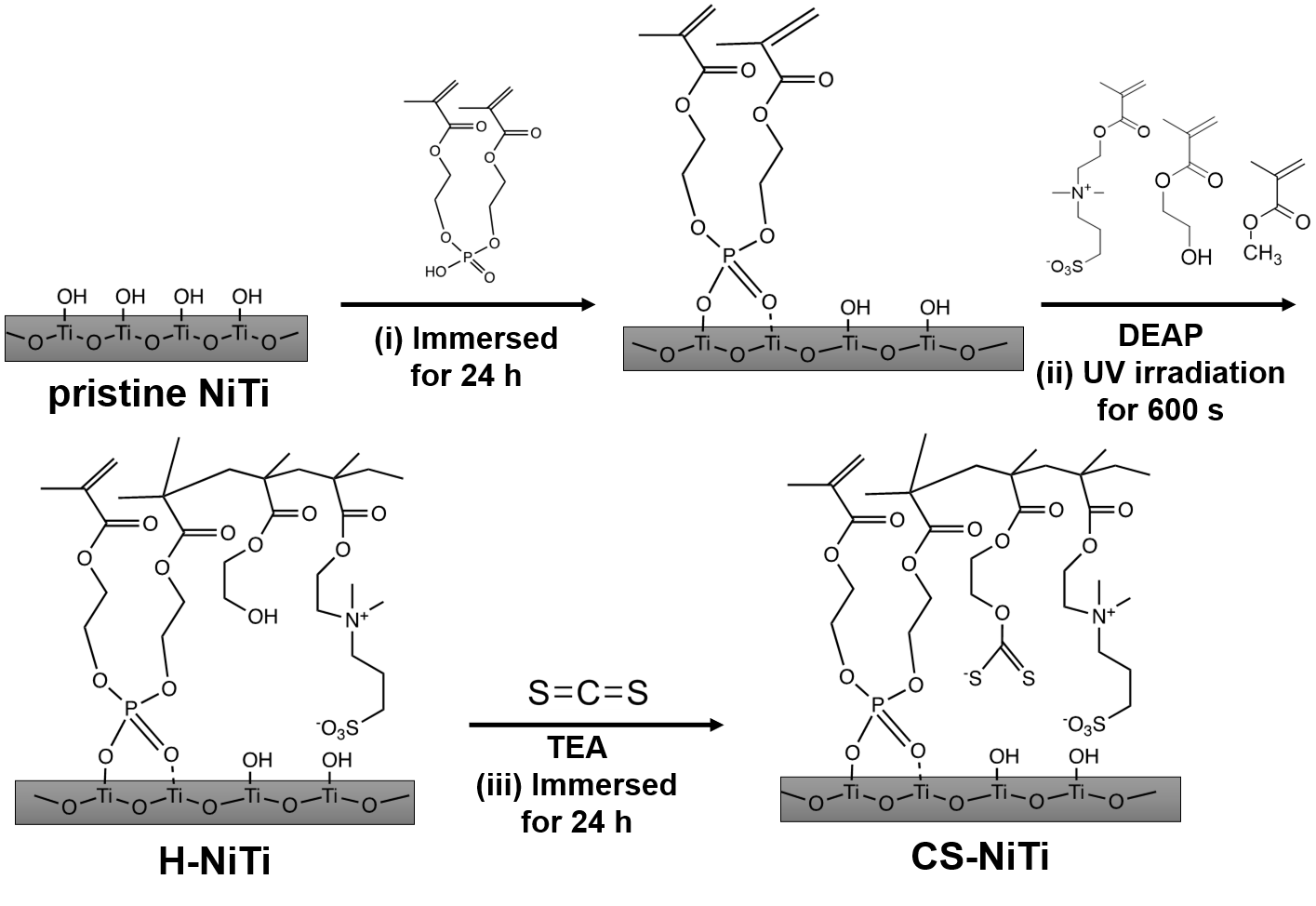


**Supplementary Figure S1** The fabrication process of CS-NiTi. (i) The surface of NiTi alloy was first treated with bis-HEMAP (5% ethanol solution) and immersed for 24 h. (ii) The synthesis of H-NiTi was accomplished through copolymerization with HEMA, DMAPS and MMA, initiated by DEAP and exposed to UV irradiation for 600 s. (iii) H-NiTi was immersed in a mixture of CS_2_ and TEA for 24 h to obtain CS-NiTi.


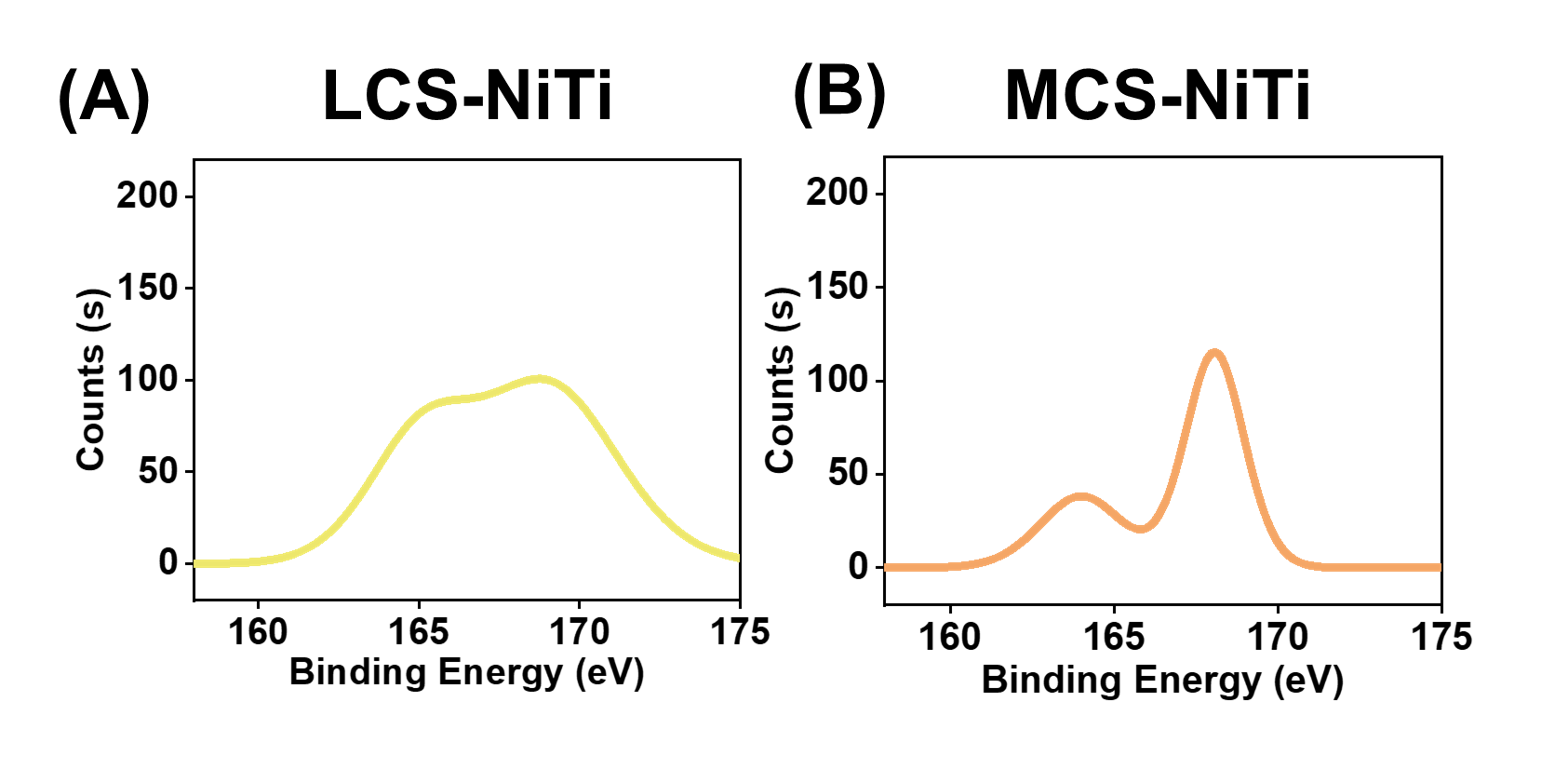


**Supplementary Figure S2** X-ray photoelectron spectroscopy analysis (XPS) for LCS-NiTi **(A)** and MCS-NiTi **(B)**.


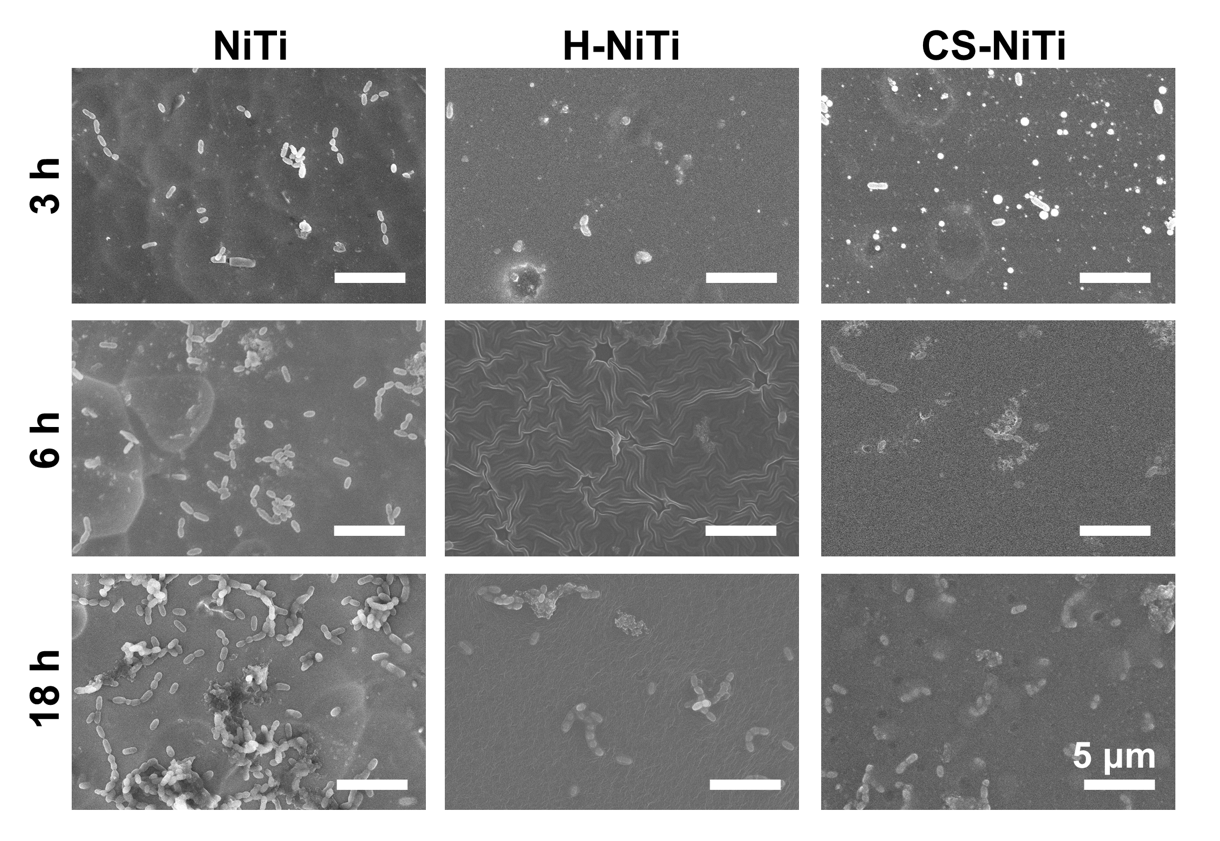


**Supplementary Figure S3** Scanning electron microscope (SEM) images of pristine NiTi, H-NiTi and CS-NiTi after being incubated with bacteria suspension for 3, 6, and 18 h.

**
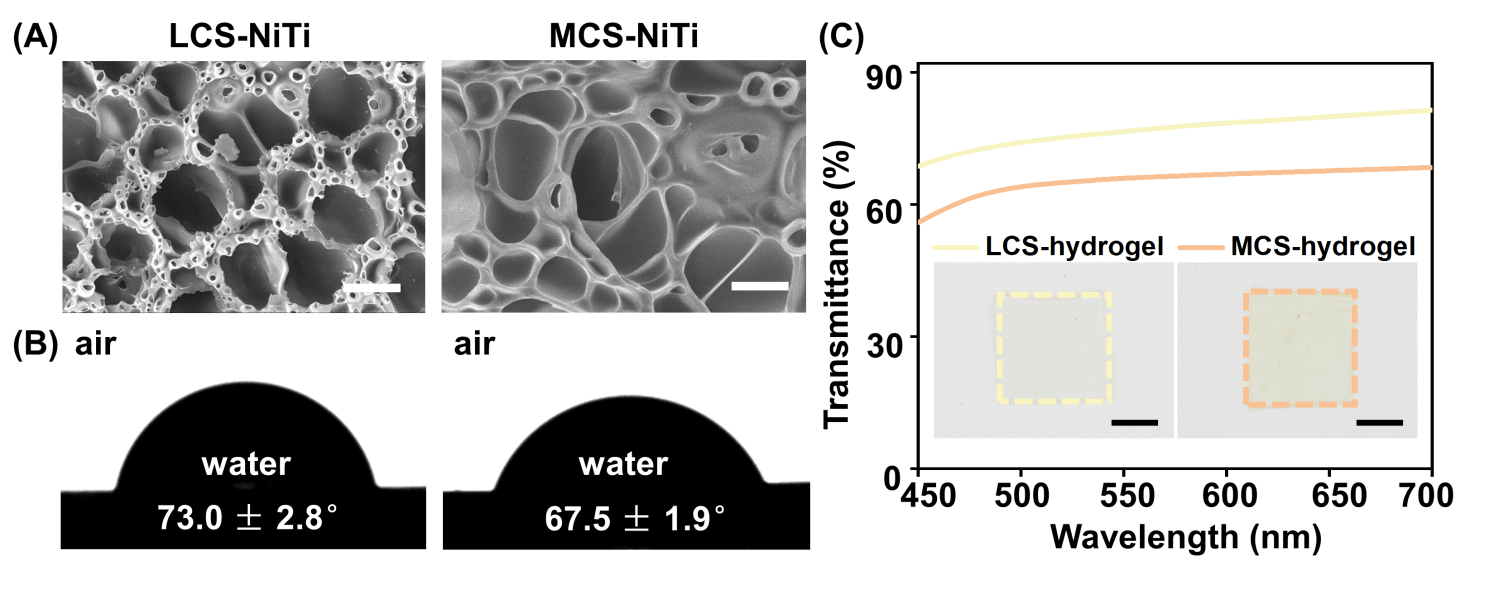
**

**Supplementary Figure S4** SEM images **(A)**, water contact angles **(B)**, and transmittance**(C)** of LCS-NiTi and MCS-NiTi. The insets in **(C)** showed the optical images of LCS-hydrogel and MCS-hydrogel. Scale bar: 10 μm for **(A)** and 5 mm for **(C)**.
